# Joint EANM-SNMMI guideline on the role of 2-[^18^F]FDG PET/CT in no special type breast cancer (endorsed by the ACR, ESSO, ESTRO, EUSOBI/ESR, and EUSOMA)

**DOI:** 10.1007/s00259-024-06696-9

**Published:** 2024-05-14

**Authors:** Sofia C. Vaz, John Patrick Pilkington Woll, Fatima Cardoso, David Groheux, Gary J. R. Cook, Gary A. Ulaner, Heather Jacene, Isabel T. Rubio, Jan W. Schoones, Marie-Jeanne Vrancken Peeters, Philip Poortmans, Ritse M. Mann, Stephanie L. Graff, Elizabeth H. Dibble, Lioe-Fee de Geus-Oei

**Affiliations:** 1 Nuclear Medicine-Radiopharmacology, Champalimaud Clinical Center, Champalimaud Foundation, Lisbon, Portugal; 2 Department of Radiology, Leiden University Medical Center, Leiden, The Netherlands; 3 PET-CT Department, Clinica Delgado AUNA, Lima, Peru; 4 Breast Unit, Champalimaud Clinical Center, Champalimaud Foundation, Lisbon, Portugal; 5 Nuclear Medicine Department, Saint-Louis Hospital, Paris, France; 6 University Paris-Diderot, INSERM U976, Paris, France; 7 Centre d’Imagerie Radio-Isotopique (CIRI), La Rochelle, France; 8 Department of Cancer Imaging, King’s College London, London, UK; 9 King’s College London and Guy’s & St Thomas’ PET Centre, London, UK; 10 School of Biomedical Engineering and Imaging Sciences, King’s College London, London, UK; 11 Molecular Imaging and Therapy, Hoag Family Cancer Institute, Newport Beach, CA USA; 12 University of Southern California, Los Angeles, CA USA; 13 Dana-Farber Cancer Institute/Brigham and Women’s Hospital, Boston, MA USA; 14 Harvard Medical School, Boston, MA USA; 15 Breast Surgical Oncology, Clinica Universidad de Navarra, Madrid, Cancer Center Clinica Universidad de Navarra, Navarra, Spain; 16 Directorate of Research Policy, Leiden University Medical Center, Leiden, The Netherlands; 17 Department of Surgical Oncology, Netherlands Cancer Institute, Amsterdam, The Netherlands; 18 Department of Surgery, Amsterdam University Medical Center, Amsterdam, The Netherlands; 19 Department of Radiation Oncology, Iridium Netwerk, Antwerp, Belgium; 20 University of Antwerp, Wilrijk, Antwerp, Belgium; 21 Radiology Department, RadboudUMC, Nijmegen, The Netherlands; 22 Lifespan Cancer Institute, Providence, Rhode Island, USA; 23 Legorreta Cancer Center at Brown University, Providence, Rhode Island, USA; 24 Department of Diagnostic Imaging, The Warren Alpert Medical School of Brown University, Providence, Rhode Island, USA; 25 Biomedical Photonic Imaging Group, University of Twente, Enschede, The Netherlands; 26 Department of Radiation Science & Technology, Technical University of Delft, Delft, The Netherlands

**Keywords:** 2-[^18^F]FDG, Breast cancer, No special type, PET/CT, EANM, SNMMI

## Abstract

**Introduction:**

There is much literature about the role of 2-[^18^F]FDG PET/CT in patients with breast cancer (BC). However, there exists no international guideline with involvement of the nuclear medicine societies about this subject.

**Purpose:**

To provide an organized, international, state-of-the-art, and multidisciplinary guideline, led by experts of two nuclear medicine societies (EANM and SNMMI) and representation of important societies in the field of BC (ACR, ESSO, ESTRO, EUSOBI/ESR, and EUSOMA).

**Methods:**

Literature review and expert discussion were performed with the aim of collecting updated information regarding the role of 2-[^18^F]FDG PET/CT in patients with no special type (NST) BC and summarizing its indications according to scientific evidence. Recommendations were scored according to the National Institute for Health and Care Excellence (NICE) criteria.

**Results:**

Quantitative PET features (SUV, MTV, TLG) are valuable prognostic parameters. In baseline staging, 2-[^18^F]FDG PET/CT plays a role from stage IIB through stage IV. When assessing response to therapy, 2-[^18^F]FDG PET/CT should be performed on certified scanners, and reported either according to PERCIST, EORTC PET, or EANM immunotherapy response criteria, as appropriate. 2-[^18^F]FDG PET/CT may be useful to assess early metabolic response, particularly in non-metastatic triple-negative and HER2+ tumours. 2-[^18^F]FDG PET/CT is useful to detect the site and extent of recurrence when conventional imaging methods are equivocal and when there is clinical and/or laboratorial suspicion of relapse. Recent developments are promising.

**Conclusion:**

2-[^18^F]FDG PET/CT is extremely useful in BC management, as supported by extensive evidence of its utility compared to other imaging modalities in several clinical scenarios.

**Supplementary information:**

The online version contains supplementary material available at 10.1007/s00259-024-06696-9.

## Objectives

The aim of this guideline is to provide information regarding the role of 2-[^18^F]fluoro-2-deoxy-D-glucose positron emission tomography/computed tomography (2-[^18^F]FDG PET/CT) in patients with no special type breast cancer (NST BC), summarizing its indications according to updated literature evidence.

This guideline should be viewed as a dynamic document in a constantly evolving field, rather than a definitive document or summary of existing protocols. Moreover, modifications may be considered according to local legislation and regulations.

## Methodology

The idea for this document was formulated by a subset of members of the EANM Oncology and Theranostics Committee. The concept of this guideline was approved by the EANM Board; then, the SNMMI was invited and named its representatives. The EANM board approved suggested experts and recommended others with the aim of creating a multidisciplinary team of experts. The final version received the comments from the EANM national societies and SNMMI public comments and was endorsed by the American College of Radiology (ACR), European Society of Surgical Oncology (ESSO), European Society for Radiotherapy and Oncology (ESTRO), European Society of Breast Imaging/European Society of Radiology (EUSOBI/ESR), and European Society of Breast Cancer Specialists (EUSOMA).

This guideline provides practical recommendations to be applied in clinical practice. All sections were written according to updated literature and state-of-the-art information and then critically verified by the writing committee.

For the writing section “Indications for 2-[^18^F]FDG PET/CT in no special type breast cancer”, the literature search was based on the international Appraisal of Guidelines, Research and Evaluation (AGREE) tool [1]. We used the 23 topics from the AGREE II reporting checklist. In summary, the following steps were taken:

- 1. Clinical indications for 2-[18F]FDG PET/CT were defined by a multidisciplinary team of experts. The clinical indications were the topics for the literature search.
- 2. Keywords were selected for each indication to search the literature in the following databases: PubMed, Embase, Web of Science, Cochrane Library, and Emcare through January 2022. Preclinical studies, case reports, images of interest, abstracts-only presented in congresses, and editorials were excluded.
- 3. Relevant papers were selected for each indication based on title and abstract. Non-English papers were excluded.
- 4. Papers were critically analyzed before being included. Additional recent publications were also included whenever they were considered important by the writing committee.
- 5. Recommendations for performing 2-[18F]FDG PET/CT were discussed in online meetings and were summarized in boxes in the “Metabolic response criteria” (Box nº 1) and “Indications for 2-[18F]FDG PET/CT in no special type breast cancer” (Box nº 2 to 8) sections.
- 6. For each summary box, the level of evidence and grade of recommendation was provided, following the National Institute for Health and Clinical Excellence (NICE) criteria (Table 1), as previously used in EANM documents [2–4], as follows:
- 7. Finally, the writing committee composed by a multidisciplinary team of experts votes on the scores for each recommendation’s level of evidence and grade of recommendation stating if they agree, disagree, or abstain.
- 8. The level of evidence, grade of recommendation, and percentage of agreement are provided for each recommendation in the respective box. The percentage of agreement was at least 85% for each recommendation.

**Table 1:** Level of evidence and grade of recommendation according to the National Institute for Health and Clinical Excellence (NICE)

Levels of scientific evidence	Type of scientific evidence
Ia	Systemic review (SR) with homogeneity of level 1 + studies
Ib	Level 1 studies
II	Level 2 studies. SR of level 2 studies
III	Level 3 studies. SR of level 3 studies
IV	Consensus, expert opinion with no explicit critical evaluation
Level 1 studies	Meet the following criteria: • Blinded comparison with a valid (“gold standard”) comparator test • Suitable range of patients
Level 2 studies	Show only one of these biases: • Non-representative population (the sample does not reflect the population in which the test will be used) • Comparison with unsuitable comparator (“gold standard”). The test to be assessed is part of the gold standard or the results of the test affects the performance of the gold standard • Non-blinded comparison • Case and control studies
Level 3 studies	Meet two or more of the criteria stated for level 2 studies
Recommendation grade	Interpretation of evidence
A	Evidence level Ia or Ib studies
B	Evidence level II studies
C	Evidence level III studies
D	Evidence level IV studies

## Introduction

According to GLOBOCAN 2020, female breast cancer (BC) was the leading cause of cancer incidence worldwide (2.3 million new cases, corresponding to 11.7% of all cancers) and it was the fifth leading cause of cancer mortality (685,000 deaths) [5].

The histology of the majority of diagnosed BC (75–80%) is no special type (NST) that corresponds to invasive ductal carcinoma (IDC) in the previous nomenclature; the second most common histology (10–15%) is invasive lobular carcinoma (ILC). Up to 5% of BC is considered “special types” due to distinctive characteristics as well as particular cellular and molecular behaviours. These “special types” include medullary, apocrine, neuroendocrine, mucinous, tubular, and metaplastic carcinomas [6–11]. BC is a heterogeneous disease with different biological subtypes depending on the expression of hormone receptors (HR), including oestrogen receptors (ER) and/or progesterone receptors (PR), and the levels of the human epidermal growth factor receptor 2 (HER2). There are four main subtypes of BC, although different classifications exist with small differences between them: Luminal A-like (HR + /HER2 − and low-grade/low proliferation), Luminal B-like (HR + /HER2 − and high-grade/high proliferation) – luminal-like correspond to 65% of cases, HER2 + (HR + or − /HER2 +) in 15–20%, and triple negative (HR − /HER2 −) in 10–15%.

BC usually disseminates loco-regionally to the ipsilateral axillary lymph nodes but can also spread loco-regionally to ipsilateral internal mammary or supraclavicular lymph nodes (up to stage N3c) [12, 13]. However, dissemination to contralateral axillary, internal mammary, or supraclavicular lymph nodes or to ipsilateral or contralateral cervical lymph nodes is regarded as distant metastasis (or stage M1). BC can potentially spread to any organ, but the most common sites are the skeleton, liver, lung, and brain. Different from NST, metastatic ILC more commonly features sclerotic bone metastases (sometimes of miliary type without FDG uptake) and metastases to the gastrointestinal tract and serosa. Factors associated with the presence of distant metastases at an earlier stage are younger age at diagnosis and triple-negative tumours [14, 15]. Each subtype has different biological behaviour in terms of survival, recurrence, and typical patterns of metastatic spread [16–19]. For example, bone metastases are more common in patients with HR + BC, whereas visceral metastases occur more often in HR- tumours [20]. In this regard, triple-negative breast cancers (TNBC) present with visceral metastases more often, predominantly intrapulmonary [17, 21, 22]. The HER2-enriched subtypes usually metastasize to the lung, liver, and brain and less often to the skeleton [23].

The 8th edition of the American Joint Committee on Cancer (AJCC) includes two staging systems: (1) the anatomic stage, which includes the characteristics of the primary tumour (T), nodal status (N), and distant metastasis (M), and is then further subdivided into clinical and pathologic anatomic stage, and (2) the prognostic stage, adding tumour grade, HR status, HER2 expression, and multigene panel testing results to the anatomic stage [12].

Currently, there is extensive evidence that 2-[^18^F]FDG PET/CT can be useful in BC management, including initial staging, assessing neoadjuvant systemic treatment response, assessing treatment response in the metastatic setting, searching for loco-regional or metastatic recurrence, and re-staging after therapy, as well as radiation therapy (RT) planning. The 2-[^18^F]FDG avidity of BC is related to the histologic type, receptor status (ER, PR, and HER2), tumour grade, proliferation index (Ki-67 index), and tumour size. It has been demonstrated that, in general, (1) NST histology has higher 2-[^18^F]FDG avidity than ILC, (2) TNBC has higher 2-[^18^F]FDG avidity than ER + tumours, and (3) grade 3 cancers have higher 2-[^18^F]FDG avidity than lower-grade malignancies [24–27]. 2-[^18^F]FDG uptake is also related to microvasculature density for delivering nutrients (and 2-[^18^F]FDG), glucose transporter 1 (GLUT1) for transportation of the tracer into the cell, hexokinase for tracer entering into glycolysis, number of viable neoplastic cells per volume, number of lymphocytes, and hypoxia-inducible factor 1-alpha (HIF-1a) upregulation of GLUT1 [28]. Consequently, high 2-[^18^F]FDG uptake correlates with tumour aggressiveness and is associated with a worse prognosis [3, 29–31]. A retrospective study reported that 2-[^18^F]FDG PET/CT was more likely to reveal unsuspected distant metastases in patients with stage III NST (22%) compared to patients with stage III ILC (11%) [32].

Considering there are limited data about 2-[^18^F]FDG specifically in the ILC subtype, the recommendations written in this document are applicable to NST. In the “Other developments and future applications” section, radiopharmaceuticals other than 2-[^18^F]FDG are referred to and those may be more useful to study patients with ILC. We recognize the need to have guideline/recommendations about the lobular subtype, and this may be a future project.

Several studies have documented 2-[^18^F]FDG PET/CT’s utility compared to other imaging modalities including bone scan and contrast-enhanced CT (ceCT). However, the superiority of 2-[^18^F]FDG PET/CT is still unclear in comparison with whole-body magnetic resonance imaging (wbMRI) [33–36].

In Table 2, we observe that baseline 2-[^18^F]FDG PET/CT enabled overall upstaging, when compared with conventional imaging modalities, in more than 19% of patients from IIB onwards: stage IIB (19%), IIIA (34%), IIIB (41%), and IIIC (35%). The percentage of stage modification due to 2-[^18^F]FDG PET findings is weaker in stage IIA patients but not negligible (13% in Table 2). Three studies from Table 2 also evaluated the percentage of upstaging based on the identification of distant metastasis only (meaning the exclusion of regional lymph node metastasis) and revealed staging modification in 10% of patients initially staged IIB, 20% in stage IIIA, 25% in stage IIIB, and 32% in stage IIIC [37–39].

These same authors showed that 2-[^18^F]FDG PET/CT can provide valuable information for nodal staging in around a quarter of patients, leading to upstaging in 17–24% of patients (mainly due to the identification of N3 disease) [37–39]. In one study, modifications included downstaging in 16% of patients [38].

A prospective and randomized clinical trial published in 2023 analyzed 369 patients with NST BC stage IIB (25%) or III (75%) staged with 2-[^18^F]FDG PET/CT or conventional imaging (bone scan, CT of the chest/abdomen and pelvis) [40]. 2-[^18^F]FDG PET/CT identified more distant metastases than conventional modalities, resulting in upstaging to stage IV for 12% more patients (23% vs 11%). Consequently, this led to changes in therapy decision and reduction in the number of patients initially considered for combined modality therapy (chemotherapy, surgical resection, and radiotherapy) aimed at curative intent [40].

As referred to above, despite the commonly reported upstaging after 2-[^18^F]FDG PET/CT due to the identification of N3 or distant metastases, Cochet et al. [38] also evaluated the percentage of downstaging. This prospective study compared staging with conventional imaging and 2-[^18^F]FDG PET/CT in 142 patients. They observed that 21% of patients were upstaged after 2-[^18^F]FDG PET/CT (including 8% whose stage changed from stage II/III to stage IV—detailed in Table 2) and 16% were downstaged (including 3% that were initially classified as stage IV, but changed to stage II/III), with high or medium impact on clinical management in 13% of patients (mainly because the intent to treat was modified from curative to palliative or vice-versa) [38].

A systematic review and meta-analysis from 2021 analyzed the impact of 2-[^18^F]FDG PET (3 studies), PET/CT (25 studies), and PET/MRI (1 study) and found a pooled 25% change in staging that resulted in an 18% change in management [41]. Literature shows a better diagnostic accuracy of 2-[^18^F]FDG PET/CT to detect distant metastases of NST BC compared to the combination of conventional imaging, due to its higher sensitivity (97–99% vs 56–75%) and specificity (95–99% vs 88–99%), as summarized in Table 3.

**Table 2:** Percentage of upstaging after 2-[^18^F]FDG PET/CT compared to conventional modalities for each initial TNM stage according to the 7th and 8th editions of the AJCC Cancer Staging Manual (adapted from [34])

			Percentage of upstaging after 2-[^18^F]FDG PET/CT								
Stage	TNM (based on conventional imaging)	Groheux et al. [37] Prospective, *n* = 254, 86% NST		Cochet et al. [38] Prospective, *n* = 142, 90% NST	Riedl et al. [14] Retrospective, *n* = 134, 92% NST	Ulaner et al. [42] Retrospective, *n* = 232 TNBC, 94% NST	Ulaner et al. [43] Retrospective, *n* = 238, 86% NST	Lebon et al. [44] Retrospective, *n* = 107, 85% NST	Ko et al. [39] Retrospective, *n* = 195, 88% NST	Overall metastasis range (mean)	Distant metastasis range (mean)
I	T1N0M0	NA		NA	5	0	NA	[8]	NA	0–8 ** (4)**	NA
IIA	T0N1M0 T1N1M0 T2N0M0	4.5 [2.3]		36 [9]	5	5	4	[11]	24 [0]	4–36 ** (13)**	2–9 **(6)**
IIB	T2N1M0 T3N0M0	16.1 [10.7]		18 [7]	17	15	14	[15]	39 [13]	14–39 **(19)**	7–13 **(10)**
IIIA	T1N2M0 T2N2M0 T3N1M0 T3N2M0	31.7 [17.5]		33 [0]	31	17	24	[44]	54 [22]	17–54 **(34)**	18–22 **(20)**
IIIB	T4N0M0 T4N1M0 T4N2M0	51.4 [36.5]		32 [21]	50	57			27 [17]	24–57 **(41)**	17–37 **(25)**
IIIC	AnyT N3M0	47.1 [47.1]		13 [13]	50	33			37 [37]	13–50 **(35)**	13–47 **(32)**

In these studies, the predominant histological type was NST BC (ranging from 84 to 95%)

Conventional modalities generally included physical examination, mammography, and/or US of the breast. Bone scan was also done in two studies [37, 38]. In one study, US of the liver, chest X-ray, or other imaging techniques were performed based on the clinical situation [38]

The values correspond to the overall upstaging rate (%), including both regional nodal metastasis and distant metastasis. The values inside square brackets correspond to distant upstaging rate only (%) (excluding regional lymph nodes)

*NA* data not available

**Table 3:** Diagnostic accuracy of 2-[^18^F]FDG PET/CT and conventional imaging to detect distant metastases of BC

	**2-[**^**18**^**F]FDG**#	**Conventional imaging**#
Sensitivity (%)	97* 99” 97.5ª	56* 57” 75.4ª
Specificity (%)	95* 95” 98.8ª	91* 88” 98.7ª
Positive predictive value (%)	95.4ª	93.4ª
Positive likelihood ratios	21”	4.8”
Negative predictive value (%)	99.4ª	94.3ª
Negative likelihood ratios	0.02”	0.49”

#In these studies, the predominant histological type was NST BC

*Hong S et al. [45]—meta-analysis evaluating distant metastases; conventional imaging modalities included CT, chest X-ray, bone scan, and ultrasonography (US)

“Sun Z et al. [46]—meta-analysis evaluating distant metastases; conventional imaging modalities included CT, chest X-ray, bone scan, and US

ªJung NY et al. [47]—retrospective study (*n* = 1161) evaluating locoregional recurrence, distant metastases, and incidental cancer; conventional imaging included mammography, US, bone scan, and chest X-ray

Overall, several studies have demonstrated a good diagnostic accuracy of 2-[^18^F]FDG PET/CT to detect distant metastases, compared to conventional imaging, in particular due to its high sensitivity (Table 2 and 3).

Additionally, 2-[^18^F]FDG PET/CT seems to play a role in the context of personalized medicine, emerging as a useful imaging modality for response assessment, allowing for early identification of non-responding tumours, providing information regarding adverse therapeutic effects, and defining the right moment to implement changes in therapeutic approach or shift to a subsequent line of treatment with benefits of disease control and cost-effectiveness [48, 49].

## 2-[^18^F]FDG PET/CT preparation and acquisition

Patient preparation should follow the “FDG PET/CT EANM procedural guidelines for tumour imaging version 2.0” and the American “Society of Nuclear Medicine and Molecular Imaging (SNMMI) procedure guideline for tumor imaging with ^18^F-FDG PET/CT 1.0” [50, 51]. Several studies have shown that the metabolic flare reaction occurs between 7 and 10 days after the start of endocrine therapy, and this effect has been observed not only with tamoxifen, but also with fulvestrant and anti-aromatases [52, 53]. Therefore, it is recommended to perform 2-[^18^F]FDG PET/CT after this time interval [53, 54]. Based on routine clinical practice, our expert consensus panel suggests performing 2-[^18^F]FDG PET/CT at least 10 days (15 days if possible) after the last dose of systemic therapy (chemotherapy or endocrine therapy) to avoid the effects of the flare phenomenon or stunning reaction. This is an expert opinion-based recommendation, and not based on scientific evidence yet. Interruption of ongoing targeted therapy for therapeutic evaluation is not recommended [55]. Because of the inflammatory effect, the recommendation is to wait at least 3 months after the end of radiotherapy to search for a recurrence in the radiotherapy field.

It is also important to report whether the patient is/was taking corticosteroids when scanned; has received growth factors; was treated with radiation therapy with specification of the treated volumes or recently underwent invasive procedures, including biopsy or surgery, because it will influence 2-[^18^F]FDG uptake; and has implications in image interpretation [50].

It is recommended that patients fast for at least 4 h before radiopharmaceutical administration and be properly hydrated, serum glucose level should be < 200 mg/dl, and patients should rest in a quiet and warm environment during the 2-[^18^F]FDG-uptake time that should last 60 min (± 5 min) before image acquisition. Concurrent ceCT imaging can be considered to improve lesion detection on CT, mainly when evaluating response to treatment in patient with metastatic disease. Additionally, it may enable easier comparative imaging with follow-up CT scans. If intravenous contrast administration is performed, kidney function and history of contrast allergy should be verified before the injection and, in such cases, measures (i.e. reinforced hydration and specific medications) should be taken according to radiological protocols [56] as well as local guidelines and regulations.

The standard 2-[^18^F]FDG PET/CT starts after bladder voiding. The patient is usually in the supine position with the arms above the head, and the acquisition includes the mid-thighs to the skull.

When RT planning is considered, and to better evaluate breasts, a flat table-top and an additional scan in the prone hanging breast position may be used in the imaging acquisition [50, 57]. It is recommended to use support devices for the arms and knees to improve patient comfort and reproducibility. It is important to remember that positioning may be modified and adapted, and analgesic medication may be offered, when necessary, to improve patient comfort.

Ideally, patients should undergo a pre-treatment PET scan (baseline study) and a scan after the end of treatment (final study), performed on the same PET scanner, to evaluate response to therapy; interim PET scan may also be helpful to direct response adapted treatment protocols [58] and should be performed with the same PET scanner used in the baseline study.

### False positive findings

The most common cause of false positive 2-[^18^F]FDG-PET findings in the breast and surrounding tissues are due to (1) benign lesions that include a wide range of diagnoses such as fibroadenoma, intraductal papilloma, as well as reactive, hyperplastic, and metaplastic processes, such as fibrocystic changes and apocrine metaplasia; (2) infection and inflammation (mastitis, fat necrosis, fungal infection, granulomatous processes, such as tuberculosis and sarcoidosis, ruptured breast implant or silicone-related reaction); (3) post-surgery (seroma, muscle uptake); and (4) physiologic (e.g. brown fat activation, lactational changes result in diffuse increased uptake in the breasts) [59, 60].

In addition, changes in metabolic activity at non-cancer sites related to cancer treatments and other medical conditions and interventions may cause imaging misinterpretation at extramammary sites. Some examples include the following: (1) recent vaccinations, particularly to COVID-19, which can result in increased uptake in axillary, subpectoral, and neck nodes; (2) bone marrow repopulation in relation to systemic therapy or granulocyte colony-stimulating factor; (3) inflammation and/or immune-related adverse events such as mucositis, colitis, pneumonitis, thyroiditis, pancreatitis, adrenalitis, and hypophysitis; (4) systemic inflammatory diseases, in particular granulomatosis, sarcoidosis, and sarcoid-like reactions; (5) osteonecrosis of the jaw; (6) fracture and post-procedural inflammation.

### False negative findings

Common causes of false negative 2-[^18^F]FDG-PET findings are as follows [26, 59, 60]: (1) small lesions measuring ≤ 10 mm (or 4–5 mm in digital PET scanners [61]), (2) low-grade tumours, (3) certain histologic subtypes (e.g. lobular, tubular, carcinoma in situ, and neuroendocrine differentiation), (4) low tumour cell density (in necrotic tissue, fibrotic scar, cystic lesions, and mucinous component), (5) artefacts (for example lesions located close to prosthetic devices, or adjacent to areas of high 2-[^18^F]FDG accumulation, such as activated brown fat or bone marrow, brain, myocardium, bladder), (6) suboptimal technique (e.g. elevated blood glucose or 2-[^18^F]FDG injection without adequate fasting), (7) PET/CT procedure (e.g. patient movement or breathing artefacts), and (8) recent or ongoing therapy.

### Incidental breast findings with 2-[^18^F]FDG uptake and additional procedures

When a 2-[^18^F]FDG-avid breast lesion is incidentally detected in a study performed for reasons other than BC, further characterization with diagnostic mammography and/or breast US should be performed because these lesions are malignant in 30–40% of cases [62, 63]. Several aetiologies have been described, including unsuspected BC, lymphoma, and metastases [59, 64–67].

In the context of breast metastases, the most common aetiologies of non-mammary cancers include haematopoietic malignancies (50%), epithelial cancers (23%), such as lung and gastrointestinal adenocarcinomas and squamous cell carcinomas, and melanomas (21%) [68].

A systematic review and meta-analysis from 2019 [63] concluded that the pooled prevalence of focal incidental breast uptake on 2-[^18^F]FDG PET/CT in women was 0.61%, and in this group of patients, the pooled prevalence of malignancy was 38.7%, with invasive ductal carcinoma being the most commonly detected cancer. In case of focal incidental breast uptake without a known correlate, irrespective of CT appearance, the patient should undergo further evaluation with physical examination, breast imaging, and possible biopsy.

### Metabolic response criteria

**Table:** 

*Summary box nº 1* • **2-[**^**18**^**F]FDG PET/CT should be reported according to PERCIST or to the EORTC PET response criteria** [69–71] *(III-92%/C-85%)* • **In patients on immunotherapy, 2-[**^**18**^**F]FDG PET/CT should be reported according to the respective EANM guidelines** [72] *(IV-100%/D-92%)* • **Quantitative features are imaging biomarkers and valuable tools for prognostication** *(I-92%/A-92%)*	

*The parenthesis contains the level of evidence—percentage of agreement/grade of recommendation—percentage of agreement for each statement.*

Metabolic therapy response assessment should be performed according to either the European Organisation for Research and Treatment in Cancer (EORTC) PET response criteria or PET Response Criteria in Solid Tumors (PERCIST) [69–71, 73]. For patients with BC undergoing immunotherapy, the recently published EANM/SNMMI/AZZSNM guideline is recommended for response assessment [72].

The use of quantitative 2-[^18^F]FDG PET/CT as an imaging biomarker has proven to be a valuable tool in treatment response assessment and prognostication [74, 75], with an important predictive role in the prognosis of patients with locally advanced or metastatic BC [58, 76–79]. Such metrics include the standardized uptake values (SUV) using either the body weight (SUVbw) or the lean body mass (SUL) for normalization, metabolically active tumour volume (MATV or MTV), and total lesion glycolysis (TLG), defined as MATV × SUVmean. In this regard, in a meta-analysis by Diao et al., metrics such as the maximum standardized uptake value (SUVmax—maximal voxel intensity in a defined volume of interest) in the primary tumour were related to a higher risk of recurrence or disease progression in this group of patients. Furthermore, SUVmax showed significant prognostic value in patients with NST [80]. Prospective studies have shown that SUV, MTV, and TLG correlated with response to treatment and prognosis [81, 82].

Pak et al*.* demonstrated in their meta-analysis that volumetric parameters obtained from 2-[^18^F]FDG PET/CT were significant prognostic factors for outcome in patients with BC. They concluded that patients with a high MTV and TLG from the primary tumour have a higher risk of adverse events and patients with a high TLG from whole-body tumour burden have a higher risk of death, therefore suggesting that these volumetric parameters should be routinely used when reporting scans [83].

However, no specific cut-off values for these metrics can be recommended currently, as these values differ widely among the data published [78]. To overcome this limitation, compliance with harmonizing standards, such as the EANM Research GmbH (EARL) or American College of Radiology (ACR)/Intersocietal Accreditation Commission (IAC) accreditation, aiming at using 2-[^18^F]FDG PET/CT as a quantitative imaging biomarker [84–86], is recommended.

## Indications for 2-[^18^f]FDG PET/CT in no special type breast cancer

Each indication contains sub-headings with updated information about the role of 2-[^18^F]FDG PET/CT. Considering that most of the papers were meta-analyses, systematic reviews, and prospective studies, recommendations were graded according to available level of evidence and scored according to NICE grading. The recommendations for performing 2-[^18^F]FDG PET/CT are summarized in boxes at the beginning of each Sect. 2-[^18^F]FDG PET/CT indications were organized in the following three parts:

A- Baseline staging
B- Assessment of treatment response
C- Assessment of recurrence

### Baseline staging

Although, 2-[^18^F]FDG avidity of BC is related to receptor status (ER, PR, and HER2), tumour grade, and proliferation index (see above), the recommendations given hereafter are valuable for NST BC whatever the BC molecular subtype and the tumour biological characteristics. In a prospective study of 254 patients, the rates of extra-axillary lymph node metastases on 2-[^18^F]FDG PET/CT were higher in grade 3 than in low-grade tumours (*p* = 0.004) and in triple-negative or HER2 + tumours compared to ER + /HER2 − tumours (*p* = 0.01) [37]. Despite the rate of distant metastases not being related to tumour grade or BC subtype, which has also been found by other studies [14, 37, 43], the location of metastases differed according to primary tumour subtype (for example, triple negative and HER2 + tumours had more extra-skeletal metastases than ER + /HER2 − tumours) [37].

Therefore, recommendations hereafter were performed according to the BC clinical stage. In this section, the clinical stage is defined according to the 8th edition of AJCC classification (Table 4). The clinical stage is based on clinical examination and locoregional imaging (breast/axilla ultrasound, mammography ± breast MRI) performed before 2-[^18^F]FDG PET/CT.

This section is divided into the following parts:

- 1-Stage I
- 2-Stage IIA
- 3-Stages IIB and III
- 4-Stage IV

**Table 4:** TNM stage grouping for breast cancer adapted from the AJCC Cancer Staging Manual [12, 87]

AJCC	TNM			Category
Stage I	T1	N0	M0	Primary operable breast cancer
Stage IIA	T0	N1	M0	
	T1	N1	M0	
	T2	N0	M0	
Stage IIB	T2	N1	M0	
	T3	N0	M0	
Stage IIIA	T3	N1	M0	
	T0	N2	M0	Locally advanced breast cancer
	T1	N2	M0	
	T2	N2	M0	
	T3	N2	M0	
Stage IIIB	T4	N0	M0	
	T4	N1	M0	
	T4	N2	M0	
Stage IIIC	any T	N3	M0	
Stage IV	any T	any N	M1	Metastatic disease

#### Stage I

**Table:** 

*Summary box nº 2* • **2-[**^**18**^**F]FDG PET/CT is not recommended in stage I** *(II-100%/B-100%)*	

*The parenthesis contains the level of evidence—percentage of agreement/grade of recommendation—percentage of agreement.*

Patients with small tumour ≤ 2cm (T1 of the TNM classification) are usually treated with primary surgery combined with sentinel node biopsy. PET has limited spatial resolution (approximately 5–6 mm) and its performance is inferior to that of the sentinel node biopsy [88].

SUVmax values are lower in stage I than in higher stages [89]. In addition, the risk of distant metastases in T1N0 disease (stage I) is very low and PET imaging may lead to false positive findings. In a multicentre study of 325 women with operable BC, 2-[^18^F]FDG PET (without a CT component) suggested distant metastases in 13 patients, only three (0.9%) were confirmed as metastatic disease and 10 (3.0%) were false positives [90]. Therefore, 2-[^18^F]FDG PET/CT is not indicated for staging patients with stage I [91].

#### Stage IIA

**Table:** 

*Summary box nº 3* • **2-[**^**18**^**F]FDG PET/CT may be useful in patients with clinical stage IIA (T1N1 or T2N0), but there is not enough strong data to recommend routine use in this subgroup** *(III-100%/C-100%*)	

*The parenthesis contains the level of evidence—percentage of agreement/grade of recommendation—percentage of agreement.*

In Table 2, the percentage of stage modification due to 2-[^18^F]FDG PET findings is weaker in stage IIA patients, but not negligible (13%). In the retrospective study by Lebon et al., distant metastases were detected by FDG-PET/CT in 15% of stage IIB patients and in 11% of stage IIA patients [44]. In another recent retrospective study, however, PET/CT demonstrated distant disease in 9.8% of stage IIB BC patients, but in only 0.8% of those with stage IIA [92]. In the study by Groheux et al., stage IIA was mainly represented by tumours classified as T2N0. PET showed pathological foci in 4.5% of women (2.3% distant metastases and 2.3% extra-axillary nodes) [37]. Larger studies in which the performance of PET is examined in subcategories of patients with T2N0 disease, such as those with large tumours (T2 > 3 cm) would be useful [93].

#### Stage IIB and stage III

**Table:** 

*Summary box nº 4* • **2-[**^**18**^**F]FDG PET/CT can be recommended for baseline staging of stage IIB (preferably before surgery) and stage III (including inflammatory BC)** *(II-100%/B-100%)* • **2-[**^**18**^**F]FDG PET/CT can be done instead of, and not in combination with, conventional imaging modalities for staging** (combination of bone scan, chest X-ray or CT-chest, and ultrasound of the liver or CT-abdomen) *(II-100%/B-100%)* • **2-[**^**18**^**F]FDG PET/CT is recommended in baseline treatment planning and may improve RT planning** *(III-100%/C-100%)*	

*The parenthesis contains the level of evidence—percentage of agreement/grade of recommendation—percentage of agreement for each statement.*

##### Role of 2-[^18^F]FDG PET/CT in baseline staging of stage IIB

Two recent papers about clinical management, including a good clinical practice guideline and a meta-analysis, which were published in 2020 and 2021, respectively, concluded that 2-[^18^F]FDG PET/CT can be recommended for initial staging to identify distant metastases in patients with clinical stage ≥ IIB BC [3, 93]. Several studies support this indication (Table 2), including a retrospective multicentre study [39], which included 195 patients with clinical stage IIA–IIIC, and compared 2-[^18^F]FDG PET/CT and conventional imaging modalities (combination of bone scan, chest X-ray or CT-chest, and ultrasound of the liver or CT-abdomen). The authors showed that 2-[^18^F]FDG PET/CT before neoadjuvant therapy enabled the identification of more extensive disease in 37% of patients, including more nodal metastases in 23% and distant metastases in 14%. Additionally, the same authors concluded that due to the high detection rate, comparable cost ($1604.37 vs $1679.94), lower radiation dose (14 mSv vs 21 mSv), and greater convenience, 2-[^18^F]FDG PET/CT should be considered as an alternative to conventional imaging modalities. Another study in 2020 revealed that 2-[^18^F]FDG PET/CT decreased the false-positive rate by 50% compared with conventional imaging and, therefore, decreased the necessity for further work-up due to incidental findings, preventing delay of treatment in a cost-effective manner [94]. In a prospective study to evaluate the role of 2-[^18^F]FDG PET/CT in the initial staging of 142 patients with ≥ T2 BC, when compared with conventional imaging modalities (mammogram and/or breast US, bone scan, abdominal US and/or CT, X-rays and/or CT of the chest), 21% of patients were upstaged by 2-[^18^F]FDG PET/CT (including 8% from stage II or III to stage IV) and 16% were down-staged (including 3% from stage IV to stage II or III) [38]. Several other studies have also highlighted the role of 2-[^18^F]FDG PET/CT in early-stage disease [74, 95], with the percentage change in staging after 2-[^18^F]FDG PET/CT by initial TNM summarized in Table 2.

2-[^18^F]FDG PET/CT may add value in the assessment of lymph node regions not easily accessible by US, including internal mammary and mediastinal lymph nodes [96]. A prospective study verified that 2-[^18^F]FDG PET/CT provided useful information in 13% of patients with clinical T3N0, T2N1, or T3N1 disease, because of the detection of extra-axillary regional lymph nodes in 6.5% and distant metastases in 9% of patients [97].

Finally, besides the higher sensitivity and specificity of 2-[^18^F]FDG PET/CT for staging of distant disease compared to combined conventional staging, performing a hybrid study in a single visit may be more convenient for the patient.

##### Role of 2-[^18^F]FDG PET/CT in baseline staging in stage III (including inflammatory BC)

According to the National Comprehensive Cancer Network (NCCN), LABC corresponds to AJCC stages IIIC, IIIB, and IIIA (except for T3N1 tumours) [98]. LABC has at least one of the following characteristics: T4 or N2 or N3 (Table 4). 2-[^18^F]FDG PET/CT has been shown to be very effective for staging of LABC and inflammatory BC (T4d).

In LABC, the percentage of patients with extra-axillary lymph node involvement detected by 2-[^18^F]FDG PET/CT varies between 10 and 29% [99–101]. Considering the prevalence of axillary lymph node involvement reaches up to 80% in this setting, the usefulness of 2-[^18^F]FDG PET/CT is related to its capability of detecting lymph node metastases [100, 102]. Nevertheless, based on the available literature, a negative axilla on 2-[^18^F]FDG PET/CT does not exclude the need for a sentinel lymph node biopsy. Moreover, studies have shown 2-[^18^F]FDG PET/CT detects distant metastases in 6–26% of patients with LABC [101, 103–105].

Furthermore, as previously mentioned, the recent prospective multicenter study by Dayes et al. [40] demonstrated that the proportion of patients initially staged as IIB and III, who were subsequently upstaged to stage IV following 2-[^18^F]FDG PET-CT, was significantly higher compared to those evaluated using conventional imaging modalities (23% vs 11%), thereby influencing treatment decisions.

Inflammatory BC is a distinct and aggressive subtype of BC characterized by rapid onset and a high propensity for metastatic disease at presentation, with approximately 80% of patients presenting with axillary lymph node involvement and 40% with distant metastases. Many guidelines recommend incorporating 2-[^18^F]FDG PET/CT in the staging of inflammatory BC [98, 106].

2-[^18^F]FDG PET/CT improves nodal staging in inflammatory BC, as demonstrated by a retrospective study correlating SUVmax of regional lymph nodes with histopathology, reporting a sensitivity of 89% and specificity of 99% [107]. Furthermore, it surpasses conventional imaging methods (including mammography, breast/regional lymph node ultrasound, whole-body bone scan, chest X-ray or CT, and abdominal ultrasound or CT) in detecting distant metastases, achieving sensitivity rates of 96–100% and specificity rates of 91–98%, compared to the combined conventional imaging sensitivity of 60–84% and specificity of 67–83% [107–114].

##### Role of 2-[^18^F]FDG PET/CT in the baseline treatment planning of stage IIB and III

In patients with BC and confirmed axillary nodal involvement, 2-[^18^F]FDG PET/CT is useful prior to surgery or neoadjuvant therapy because it identifies distant metastases ranging from 6 to 26% of cases [4]. Furthermore, it is useful for patient selection and planning of RT volumes.

In a prospective study from 2021, patients with high-risk primary BC were staged with 2-[^18^F]FDG PET/CT. Distant metastases were identified in 23% of patients, and more advanced loco-regional disease in 16% [115]. This led to more extensive RT and stage modification in 40% of patients [115]. Therefore, the authors concluded that 2-[^18^F]FDG PET/CT should be considered for initial staging in high-risk primary BC to improve treatment planning.

Another prospective trial evaluated the diagnostic performance of pre-treatment 2-[^18^F]FDG PET/CT and its impact on RT in patients with LABC [116]. The authors observed 2-[^18^F]FDG PET/CT detected 20% more distant metastatic disease or nodal disease outside conventional RT fields not visualized on conventional imaging (including mammography and breast US, bone scan, and CT chest, abdomen, and pelvis scans), leading to a change in RT plan [116].

2-[^18^F]FDG PET/CT changed regional RT planning in patients with involved axillary lymph nodes in around 20% of cases [117, 118]. Also, it improved the RT procedure of involved internal mammary lymph nodes, allowing for conformal 3-dimensional (3D) planning and dose-escalation [119, 120].

Regarding inflammatory BC, 2-[^18^F]FDG PET/CT led to a change in RT plan in 18% of patients and in overall treatment in approximately 10% of patients [121]. Other authors stated that 2-[^18^F]FDG PET/CT changed the initial staging and, consequently, the treatment strategy in up to 50% of patients with inflammatory BC [4, 122]. Pre-treatment 2-[^18^F]FDG PET/CT has also provided valuable information on disease extent evaluation, leading to changes in post-mastectomy RT target volumes and radiation doses in approximately 20% of patients [113, 123].

#### Stage IV

**Table:** 

*Summary box nº 5* • **2-[**^**18**^**F]FDG PET/CT can be useful for determining the extent of metastatic disease (outside the brain) and improving treatment planning** *(III-100%/C-100%)* • **2-[**^**18**^**F]FDG PET/CT can be done instead of, and not in addition to separate conventional imaging modalities** (combination of bone scan, chest X-ray or CT-chest, and ultrasound of the liver or CT-abdomen) *(II-100%/B-100%)*	

*The parenthesis contains the level of evidence—percentage of agreement/grade of recommendation—percentage of agreement for each statement.*

The presence, extent, and localization of distant metastases are key prognostic factors in BC patients and play a central role in therapeutic decision-making. Studies have not focused on patients with known stage IV disease, but on patients with LABC, in whom 2-[^18^F]FDG PET/CT has shown to perform better than bone scans or CT scans in assessing metastatic disease.

In the study from Choi et al. [124], encompassing a group of 154 consecutive patients, the sensitivity and specificity in detecting distant metastasis was 61.5% and 99.2%, respectively, for conventional imaging, and 100% and 96.4%, respectively, for 2-[^18^F]FDG PET/CT.

The bone is the most frequent site of metastases in patients with BC. In the study from van Es et al. [125], baseline ceCT, bone scan, and 2-[^18^F]FDG PET for all patients included in the IMPACT-MBC study were reviewed for bone lesions. In total, 3473 unequivocal bone lesions were identified in 102 evaluated patients (39% by ceCT, 26% by bone scan, and 87% by 2-[^18^F]FDG PET). Additional bone lesions on 2-[^18^F]FDG PET plus ceCT compared with bone scan plus ceCT led to a change in MBC management recommendations in 16% of patients [125].

Several other studies showed that bone scan is not useful when 2-[^18^F]FDG PET/CT is performed [37, 111, 126]. In particular, PET/CT was more sensitive and more specific than bone scan or ceCT for detecting lytic or mixed bone metastases and bone marrow involvement [126]. 2-[^18^F]FDG uptake was more variable in osteoblastic metastases, and careful reading of CT data from PET/CT may help detect them [13]. In a study of 23 BC patients with bone metastases, PET/CT detected more lesions than bone scan (mean, 14.1 vs. 7.8 lesions, respectively, *p* < 0.01) [127]. [^18^F]NaF PET/CT can also be of added value in the case of sclerotic bone lesions [128] (see “Other radiopharmaceuticals” section).

2-[^18^F]FDG PET/CT also performs well in detecting distant lymph nodes, pleural, hepatic, splenic, adrenal, and pelvic metastases. In 117 patients with LABC, 2-[^18^F]FDG PET/CT detected distant metastases in 43 patients (37%) [111]. The sensitivity and specificity of PET/CT were 100% and 97.7% for the detection of bone lesions (compared with 76.7% and 94.2%, respectively, for planar bone scan); 100% and 99.1%, respectively, for the detection of pleural metastases (vs. 50% and 100% for dedicated CT); and 85.7% and 98.2%, respectively, for the detection of pulmonary metastases (vs. 100% and 98.2% for dedicated chest CT) [111]. In this study, PET was therefore less sensitive than chest CT for the detection of small lung nodules, which could be explained by the partial volume effect and respiratory motion. Regarding distant lymph node involvement, 2-[^18^F]FDG PET/CT detected supra-diaphragmatic distant lymph nodes in 18 patients and infra-diaphragmatic nodes in four patients. Among 117 patients, 10 were diagnosed with liver metastases, and 2-[^18^F]FDG PET/CT confirmed the nine cases detected by abdominal CT and/or liver ultrasound and enabled the identification of one additional patient [111].

### Assessment of treatment response

#### Assessment of neoadjuvant treatment response in non-metastatic breast cancer

**Table:** 

*Summary box nº 6* • 2**-[**^**18**^**F]FDG PET/CT may be used to assess early metabolic response in non-metastatic breast cancer, particularly in TNBC and HER2 + ***(II-100%/ B-100%)*	

*The parenthesis contains the level of evidence—percentage of agreement/grade of recommendation—percentage of agreement.*

Considering 2-[^18^F]FDG PET/CT provides different information depending on timing and therapeutic procedure, the following paragraphs describe its potential use in the early evaluation of metabolic response in patients under neoadjuvant therapy, as well as response evaluation after completion of it. While results are promising, further evidence is needed to support this recommendation.

##### Metabolic response at early response evaluation in the primary tumour to primary systemic therapy

2-[^18^F]FDG PET/CT is associated with good sensitivity, but lower specificity, to predict early histopathological response to neoadjuvant therapy (as early as the first cycle of treatment), independent of BC subtypes. Metabolic changes after systemic therapy, measured by 2-[^18^F]FDG PET/CT, usually predict treatment response earlier than anatomic changes [3, 58]. Many clinical trials found the ability to distinguish responding from non-responding tumours, with the potential for personalized adaptation of therapy courses [55, 58, 129, 130].

In four meta-analyses, regrouping 920 [131], 781 [132], 745 [133], and 1119 [134] patients, PET sensitivity and specificity in predicting the pathological response early were respectively 84% and 66% in the first, 84% and 71% in the second, 81% and 79% in the third, and 82% and 79% in the fourth meta-analysis. Overall, the sensitivity was in the order of 80–85% and the specificity was somewhat lower. A fifth meta-analysis, comparing PET to MRI to predict pCR, showed that PET was more sensitive and MRI more specific [135]. Finally, a most recent meta-analysis of 1630 patients (assessing interim and post-treatment 2-[^18^F]FDG PET scans) suggested that the use of 2-[^18^F]FDG PET or PET/CT for evaluation of response to neoadjuvant treatment provides significant predictive value for disease recurrence and survival in BC patients [136].

Overall, these meta-analyses also highlighted large disparities between studies. In most studies, a cut-off for the reduction in the primary tumour SUV_max_ value (ΔSUV_max_) has been used to discriminate metabolic responders (decrease in SUV above the threshold) from non-responders. The cut-off value that offers the best prediction of pathological complete response (pCR) at the end of the neoadjuvant treatment has most often been determined from the area under the receiver operating characteristic (ROC) curves analysis. Unfortunately, the optimal cut-off varied between the studies. The tumour subtype and the treatment used should be considered to assess response in patients with BC treated by neoadjuvant treatment. Triple-negative tumours have higher FDG uptake than the others. The type of treatment is also crucial. In triple-negative tumours, a team observed that the SUV decreased significantly more with dose-dense and dose-intense treatment than with conventional dose chemotherapy regimen [137]. An ancillary study in the NeoALTTO trial showed that SUV reduced more with lapatinib + trastuzumab than with trastuzumab alone [55].

##### Early assessment of neoadjuvant treatment in triple-negative tumour

Pathological complete response (pCR) is associated with better survival and is therefore the objective in patients with TNBC [138]. Several studies evaluated the value of 2-[^18^F]FDG PET for early prediction of pCR in patients with TNBC [137, 139–145]. In 78 patients with TNBC, the change in SUV_max_ after two cycles of neoadjuvant chemotherapy was strongly correlated with pCR at surgery. [137]. These patients were followed up and the risk of recurrence was greater when the SUV_max_ of the primary tumour did not decrease or only slightly decreased after two cycles of neoadjuvant treatment [137].

##### Early assessment of neoadjuvant treatment in HER2 overexpressing tumour

TBCRC 026 [146] evaluated patients with stage II or III HER2 + BC for reduction of metabolic activity under neoadjuvant treatment. 2-[^18^F]FDG PET/CT was performed at baseline and 15 days after therapy initiation of pertuzumab and trastuzumab. The difference between the groups who obtained pCR at the end of the neoadjuvant treatment and those who did not was documented by a median percent reduction in SULmax (63.8% vs 41.8%) and SULmax reduction ≥ 40% (83% vs 52%).

The multicentre randomized phase 2 study AVATAXHER included 142 patients who initially received standard therapy combining docetaxel and trastuzumab and evaluated the role of 2-[^18^F]FDG PET in modulating neoadjuvant treatment according to the metabolic response after one cycle [129]. In the case of poor response after one cycle (low or absence of SUV decrease), a randomization was performed: arm A received bevacizumab in addition to the initial treatment starting from cycle-3, while arm B continued the initial treatment. At treatment completion, the pCR rates were respectively 37/69 (53.6%) for responding patients (high SUV decrease after one cycle), 21/48 (43.8%) for arm A, and 6/25 (24.0%) for arm B. Thus, a change in treatment led to an increase in the pCR rate in poor responders. Unfortunately, long-term follow-up of this patient cohort showed that improvement of pCR did not modify disease-free survival [147].

More recently, in the PHERGain multicentre, randomized, open-label, non-comparative, phase 2 study, 356 patients with HER2 + early-stage BC were included [130, 148]. 2-[^18^F]FDG PET identified patients who were likely to benefit from chemotherapy-free dual HER2 blockade with trastuzumab and pertuzumab and a reduced negative impact on global health status [130].

##### Early assessment of neoadjuvant treatment in HR-positive tumour without HER2 overexpression

The majority of HR + tumours have weak 2-[^18^F]FDG uptake. The chemosensitivity of these tumours is variable but limited and pCR is rarely reached [149, 150]. In the study from Humbert et al. [141], evaluating the predictive value of 2-[^18^F]FDG PET by BC subgroups, pCR was obtained in only one of the 53 patients with a luminal BC. Therefore, 2-[^18^F]FDG PET/CT seems to have limited value to predict pCR in this subgroup.

##### Metabolic response after primary systemic therapy

A 2020 meta-analysis [136] included 17 studies assessing interim and post-treatment 2-[^18^F]FDG PET scans and described that the pooled hazard ratio of metabolic responses on disease-free survival and OS was 0.21 and 0.20 for interim PET scans and 0.31 and 0.26 for post-treatment PET scans, respectively. However, several studies have shown that 2-[^18^F]FDG PET is not very sensitive at the end of treatment to reveal the residual primary tumour tissue [151, 152]. 2-[^18^F]FDG PET/CT shows a tendency toward underestimation of the residual tumour [153]; therefore, MRI performs better in this context [151, 154].

Two systematic reviews [155, 156] analyzed the diagnostic performance of clinical examination, axillary US, breast MRI, and whole-body 2-[^18^F]FDG PET/CT and found that, currently, there is no accurate non-invasive technique to identify patients with a complete axillary response after neoadjuvant therapy. Given the ongoing debate about optimal axillary management after neoadjuvant therapy, sentinel lymph node biopsy with or without the removal of marked initially positive lymph nodes should be considered in patients with an imaging-based negative axilla.

However, at the end of neoadjuvant chemotherapy, 2-[^18^F]FDG PET/CT can be useful to perform a whole-body examination, to exclude metabolically active regional lymph nodes or distant metastases before breast surgery.

#### Assessing treatment response in metastatic breast cancer

**Table:** 

*Summary box nº 7* • **2-[**^**18**^**F]FDG PET/CT may play a role in monitoring treatment response in metastatic breast cancer** *(III-100%/C-85%)* • **2-[**^**18**^**F]FDG PET/CT may be particularly useful to assess bone metastases and enable early response to treatment evaluation** *(III-100%/C-100%)*	

*The parenthesis contains the level of evidence—percentage of agreement/grade of recommendation—percentage of agreement.*

Several studies have demonstrated the usefulness of 2-[^18^F]FDG PET/CT to assess response to systemic therapy in metastatic BC, and some have shown its superiority compared to CT, including a systematic literature review from 2019 and a prospective clinical study from 2023 [157–160].

In patients with multiple distant metastases, 2-[^18^F]FDG PET/CT allows for an earlier detection of progression, when compared with conventional imaging, enabling a change in treatment with a potential impact on survival [161]. The results of a prospective clinical study (MESTAR) of 87 patients with metastatic BC revealed that disease progression was detected first by 2-[^18^F]FDG PET/CT in half of the patients, with a mean time of 6 months earlier than on ceCT [159]. Furthermore, tumour response on 2-[^18^F]FDG PET/CT was significantly associated with progression-free survival and disease-specific survival [160]. A retrospective study of 300 patients with metastatic BC (86% presented with multiple metastases) verified that 2-[^18^F]FDG PET/CT improved patient management with a survival benefit of 14–24 months when 2-[^18^F]FDG PET/CT was used alone or in combination with CT to evaluate treatment response [162].

In the specific context of bone metastases, decreased 2-[^18^F]FDG uptake and increased bone sclerosis on CT images are predictors of good response to therapy [163]. Several retrospective studies have reported that 2-[^18^F]FDG PET/CT is superior to CT or bone scan to assess response to therapy [164, 165]. A prospective study of 31 patients verified an overall similar diagnostic performance between 2-[^18^F]FDG PET/CT and wbMRI [166]. However, contrary to MRI, 2-[^18^F]FDG uptake was associated with progression-free survival [166]. A prospective study of 23 women with biopsy-proven ER-positive bone-only or bone-dominant metastatic BC supported the usefulness of the early 2-[^18^F]FDG PET/CT evaluation (4 weeks after starting new endocrine therapy) to predict treatment failure. The authors reported that patients with good response on early 2-[^18^F]FDG PET/CT evaluation tend to have a longer progression-free survival, OS, and time to skeletal-related events, compared with non-responders [167]. The results of this study informed the inclusion of a 4-week time point in the ongoing phase 2 ECOG-ACRIN 1183 (FEATURE) trial.

### Assessment of recurrence

**Table:** 

*Summary box nº 8* • **2-[**^**18**^**F]FDG PET/CT is useful to detect the site and extent of recurrence when conventional imaging methods are equivocal** *(I-85%/A-100%)* • **2-[**^**18**^**F]FDG PET/CT can be recommended:** o **In patients with signs or symptoms suggestive of metastatic disease** *(I-92%/A-100%)* o **In patients with rising serum tumour markers** *(II-92%/B-85%)* o **To guide site of biopsy** *(IV-100%/D-100%)* o **To improve RT planning** *(III-100%/C-100%)* • **2-[**^**18**^**F]FDG PET/CT can substitute for CT and/or bone scan in the detection of bone metastases** *(II-100%/B-100%)*	

*The parenthesis contains the level of evidence—percentage of agreement/grade of recommendation—percentage of agreement for each statement.*

2-[^18^F]FDG PET/CT was compared prospectively with thoraco-abdominal ceCT and bone scan (all scans were performed within approximately 10 days) in 100 patients with suspected BC recurrence [168]. Twenty-two percent of patients were diagnosed with distant recurrence, 19% were classified as having local recurrence only, and in 59%, no recurrence was found. For distant recurrence, the area under the ROC curve was 0.99 for 2-[^18^F]FDG PET/CT, 0.84 for ceCT, and 0.86 for the combination of ceCT and bone scan [168]. Other guidelines suggest 2-[^18^F]FDG PET/CT may be useful to detect the site of recurrence when conventional imaging methods provide equivocal results [98, 169].

#### Locoregional recurrence

Radiological techniques are the standard imaging modalities for assessing locoregional recurrence, and, in particular, breast MRI plays an important role. 2-[^18^F]FDG PET/CT is particularly useful in the differentiation of post-treatment fibrosis from viable tumour tissue [98]. It may identify isolated loco-regional lesions [98, 169], particularly in aberrant lymph drainage locations due to previous surgery and/or RT, enabling targeted treatment.

2-[^18^F]FDG PET/CT impacted the clinical management of recurrent locoregional disease in 51–69% of patients [170].

#### Distant recurrence

High diagnostic accuracy of 2-[^18^F]FDG PET/CT for metastases identification has been shown prospectively, with an area under the ROC curve of 0.99 for 2-[^18^F]FDG PET/CT, 0.84 for thoraco-abdominal ceCT, and 0.86 for the combination of ceCT and bone scan [168]. Even in patients with early-stage BC, but presenting symptoms suspicious for first distant metastases, 2-[^18^F]FDG PET/CT was evaluated prospectively against biopsy-verified local recurrence in 225 women. 2-[^18^F]FDG PET/CT showed high diagnostic accuracy in detecting recurrence with a sensitivity, specificity, and area under the ROC curve of 1.00, 0.88, and 0.98, respectively [171].

Considering bone lesions, 2-[^18^F]FDG PET/CT allows earlier detection of bone metastases and identifies non-measurable lesions by morphologic examinations [48]. Its improved sensitivity may be due to the capability of detecting rapid osteolytic growth, as well as metastatic tumour cells within the bone marrow, before there is sufficient osteoblastic reaction detectable by bone-specific tracers or on CT imaging [170]. Furthermore, lesion-based sensitivity of 2-[^18^F]FDG PET/CT is comparable to the combination of bone scan and low-dose CT (98.2% vs. 98.6%, respectively), but is significantly higher than low-dose CT (80%) or bone scan (76%) alone [172]. Therefore, it is an equivalent substitution for CT and/or bone scan, meaning that if bone metastases are detected on 2-[^18^F]FDG PET/CT, the bone scan is not needed for confirmation [98, 173, 174].

A retrospective study of patients with suspicion of BC relapse verified that, when compared with conventional imaging (including mammography, CT, MRI, and bone scintigraphy), 2-[^18^F]FDG PET/CT changed treatment modality or intent in 48% and led to modifications in the treatment regimen (namely the RT volume or dose fractionation) in 9% [175].

Several studies, including a meta-analysis [176], have demonstrated that when there are equivocal results or findings suspicious for recurrence on conventional imaging (CT, MRI, ultrasound, bone scan, and mammography) or when tumour markers (cancer antigen 15.3, CA15.3, or carcinoembryonic antigen, CEA) increase during follow-up, the inclusion of 2-[^18^F]FDG PET/CT in the diagnostic algorithm of BC relapse changes clinical management in 40–50% of patients. The pooled sensitivity, specificity, positive likelihood ratio, negative likelihood ratio, diagnostic odds ratio, and AUC of 2-[^18^F]FDG PET or PET/CT to detect recurrent disease are 0.90, 0.81, 4.64, 0.12, 46.52, and 0.94, respectively [177]. 2-[^18^F]FDG PET/CT has also demonstrated a high PPV (0.97) and accuracy (0.83–0.86) in patients with suspected recurrent disease because of increased CA15.3 or CEA [178, 179]. In 561 consecutive patients, the median CA 15.3 value was 35.0 U/mL in cases in which no distant metastases were detected, and it was 58.9 U/mL in cases in which metastases were detected by 2-[^18^F]FDG PET/CT (*p* < 0.001) [180]. The median CEA value was 6.6 U/mL in cases without metastases and 12.4 U/mL in cases with metastases (*p* < 0.001) [180]. It should be noted that in cases of clinical suspicion, 2-[^18^F]FDG PET/CT can also reveal recurrence even in cases of negative tumour markers [181, 182]. In the case of a known recurrence (identified with clinical examination and/or conventional imaging), 2-[^18^F]FDG PET/CT is also useful to determine whether the recurrence is isolated or to classify it as either oligo- or multi-metastatic disease. Additionally, 2-[^18^F]FDG PET/CT plays a role to guide the site of biopsy in a variety of organs, as it increases the detection rate and improves diagnostic accuracy [183].

## Summary of indications for 2-[^18^F]FDG PET/CT in no special type breast cancer

In baseline staging, 2-[^18^F]FDG PET/CT plays a role from stage IIB through stage IV. 2-[^18^F]FDG PET/CT is possibly useful in patients with clinical stage IIA (T1N1 or T2N0), but there are not enough data to recommend its routine use.

Whenever possible, quantitative features (such as SUV, MTV, and TLG) should be evaluated and included in the report, because there is robust evidence indicating that these are important imaging biomarkers and valuable prognostic parameters.

When assessing response to therapy, 2-[^18^F]FDG PET/CT should be performed on EARL or ACR/IAC certified PET/CT scanners, and scans should be reported either according to PERCIST, EORTC PET response criteria, or EANM immunotherapy response criteria, as appropriate. 2-[^18^F]FDG PET/CT may be useful to assess early metabolic response, particularly in non-metastatic triple-negative and HER2 + tumours.

2-[^18^F]FDG PET/CT is also useful to detect the site and extent of recurrence when conventional imaging methods are equivocal and when there is clinical and/or laboratorial suspicion of relapse.

The concise summary of the 2-[^18^F]FDG PET/CT indications according to the clinical scenario defined in this guideline is presented in Table 5 below. Recommendations with level of evidence/grade of recommendation I/A or II/B are highlighted in bold. The remaining recommendations, particularly the ones scored as III/C need further investigation. In our opinion, it would be particularly useful to have more evidence about the role of 2-[^18^F]FDG PET/CT in assessing response to therapy in the metastatic setting and in RT planning in different clinical scenarios. We consider this the first attempt to define and organize 2-[^18^F]FDG PET/CT indications for patients with NST BC, but future work is needed to clarify scenarios still lacking robust scientific evidence.

**Table 5:** Summary of the 2-[^18^F]FDG PET/CT recommendations according to the clinical scenario

Clinical scenario		2-[^18^F]FDG PET/CT recommendations * (level of evidence/grade of recommendation)*
Baseline staging	Stage I	•** Not recommended** ***(II/B)***
	Stage IIA	• May be useful in clinical stage IIA (T1N1 or T2N0), but there is not enough data *(III/C*)
	Stage IIB and stages III	•** Baseline staging of stage IIB (preferably before surgery) and stage III (including inflammatory BC)** ***(II/B)*** •** 2-[**^**18**^**F]FDG PET/CT can be done instead of, and not in combination with, conventional imaging modalities for staging** ***(II/B)*** • Baseline treatment planning may improve RT planning *(III/C)*
	Stage IV	• Can be useful for determining the extent of metastatic disease (outside the brain) and improving treatment planning *(III/C)* **• 2-[**^**18**^**F]FDG PET/CT can be done instead of, and not in addition to separate conventional imaging modalities** ***(II/B)***
Assessment of treatment response	Non-metastatic breast cancer	**• May be used to assess early metabolic response, particularly in TNBC and HER2 +** ***(II/B)***
	Metastatic breast cancer	• May play a role in monitoring treatment response *(III/C)* • May be particularly useful to assess bone metastases and enable early response to treatment evaluation *(III/C)*
Assessment of recurrence		**• Useful to detect the site and extent of recurrence when conventional imaging methods are equivocal** ***(I/A)*** **•** 2-[^18^F]FDG PET/CT can be recommended: o** In patients with signs or symptoms suggestive of metastatic disease** ***(I/A)*** o** In patients with rising serum tumour markers** ***(II/B)*** o To guide the site of biopsy *(IV/D)* o To improve RT planning *(III/C)* • **Can substitute CT and/or bone scan in the detection of bone metastases** ***(II/B)***

## Other developments and future applications

### Other radiopharmaceuticals

While 2-[^18^F]FDG has by far been the most widely utilized PET agent, multiple other radiotracers have an impact or might have a future impact on the care of patients with BC.

#### 16α-18F-Fluoro-17β-fluoroestradiol ([^18^F]FES)

[^18^F]FES is a radiolabeled oestrogen analogue that binds to ER. It enables non-invasive, whole-body, and rapid study of functional ER on one single imaging examination. It shows a good correlation with ER immunohistochemistry and enables the detection of ER-positive tumours. [^18^F]FES was FDA-approved in 2020 as a diagnostic agent for the detection of ER-positive lesions as an adjunct to biopsy in patients with recurrent or metastatic BC. Its sensitivity is high in the bone, lymph nodes, and brain. However, liver evaluation is limited due to its physiologic biliary excretion.

The recently published Appropriate Use Criteria for [^18^F]FES endorses the following as the most appropriate uses of [^18^F]FES: (1) to assess ER functionality when endocrine therapy is considered either at initial diagnosis of metastatic BC or after progression of disease on endocrine therapy, (2) to assess the ER status of lesions that are difficult or dangerous to biopsy, and (3) to assess the ER status of lesions when other tests are inconclusive [184]. Additional clinical scenarios which may be appropriate to use [^18^F]FES include systemic staging of ILC and low-grade NST BC and subtypes of BC which may have low 2-[^18^F]FDG uptake.

#### [^18^F]Sodium fluoride (NaF)

NCCN Guidelines continue to allow for [^18^F]NaF PET to be utilized *in lieu* of bone scan with single photon agents, because the tracer uptake is based on the same principle but has a higher sensitivity and resolution, providing 3D information [98]. A prospective study of 28 patients with bone-dominant MBC evaluated before changing therapy and 4 months later concluded that change in 2-[^18^F]FDG parameters predicted time to skeletal-related events (tSRE) and time to progression (TTP), but not OS [185]. Change in [^18^F]NaF PET/CT parameters was associated with OS; however, it was not useful for predicting TTP or tSRE [185]. Further strong studies are needed to compare both radiopharmaceuticals.

#### [^18^F]Fluciclovine (FACBC)

FDG may have reduced sensitivity for primary and metastatic ILC [28, 32, 186, 187], and there is preliminary evidence that metabolic imaging with amino acid agents such as FACBC may be more sensitive than glucose agents in this setting [188–190].

#### Fibroblast activation protein inhibitor (FAPI)

Fibroblast activation protein (FAP) has been found to be overexpressed in cancer-associated fibroblasts of multiple cancer types. PET tracers targeting FAP have been utilized for imaging patients with BC with retrospective evidence that they may outperform FDG in some patients [191–194].

#### Human epidermal growth factor receptors (HER)

HER-targeted agents provide another opportunity for PET to guide targeted therapy. HER2-targeted therapies can prolong survival in patients with HER2 + malignancies [195]; however, spatial and temporal heterogeneity of HER2 may lead to suboptimal use of these therapies [196, 197]. HER2-targeted PET imaging with radiolabeled antibodies and antibody fragments has demonstrated the ability to help select patients that may best benefit from HER2-targeted therapies [198–204], even among patients with tumours previously presumed to lack HER2 expression [205]. Recent work demonstrates that patients with low HER2 expression on immunohistochemistry, but previously classified HER2-, may respond to newer HER2-targeted therapies [206, 207]. The successful treatment of HER2-low breast cancer raises new opportunities and areas of investigation for HER2-targeted imaging.

### PET/MRI

In most soft tissues, the sensitivity and specificity of CT are outperformed by MRI. In BC staging, MRI is superior to CT for detecting lymph node involvement as well as brain, bone, and liver metastases [208–210]. The information that can be obtained with MRI is not only anatomic in nature, but also allows functional evaluation. Enhancement patterns, for example, provide insight in perfusion characteristics, whereas diffusion-weighted imaging provides insight into the free movement of water molecules (Brownian motion), which in cancer is usually related to the cellular density of lesions.

PET/MRI aims to fuse the functional information of PET with the functional and anatomic information obtained with MRI. Attenuation correction is done by segmenting the body in different structures (soft tissue, fat, bone, lung), although the exact procedures are vendor and protocol-specific [211]. Still, quantification of PET signals in PET/MRI is more cumbersome than from PET/CT [212, 213], and measurement of SUVs is therefore less robust, although a strong correlation has been shown in BC metastases [214]. Furthermore, PET/MRI is still time-consuming compared to standard MRI. For evaluation of the breasts, PET/MRI can best be performed in the prone position, using a dedicated breast coil. However, studies have thus far only suggested a modest increase in specificity compared to MRI alone for evaluation of the primary tumour [215–217]. Some authors have suggested that the combination of both modalities may improve the diagnostic performance to assess local BC pCR after neoadjuvant therapy [135].

For nodal staging 2-[^18^F]FDG PET/MRI outperforms MRI alone [218, 219] and is, in a meta-analysis, similar to 2-[^18^F]FDG PET/CT [220]. Due to the higher sensitivity of MRI for bone and soft tissue lesions, early studies showed an improved sensitivity of 2-[^18^F]FDG PET/MRI over PET/CT for hepatic and bone metastases [221, 222]. Currently, CT still outperforms MRI for the evaluation of lung parenchyma, and consequently, assessment of lung metastases is more difficult with PET/MRI than with PET/CT.

A meta-analysis showed excellent diagnostic performance of 2-[^18^F]FDG PET/MRI for both nodal staging (sensitivity 94%, specificity 90%) and distant staging (sensitivity 98%, specificity 96%) [223]. According to the currently available preliminary data, PET/MRI can safely be used as an alternative to PET/CT for current indications. The improved multi-factorial functional information that can be obtained with PET/MRI potentially further characterizes cancerous lesions, which might in turn lead to more tailored therapies [224, 225]. However, these approaches, whether based on clinical evaluation or radiomics, have currently not yet left the research domain, and their impact in clinical practice remains to be proved.

### PET LINAC

Image-guided radiation therapy (IGRT) entails RT delivery guided by images to verify patient positioning, conventionally using X-ray imaging. With the transition from field-based RT to volume-based RT, more advanced techniques became available, including cone-beam CT scanning. This allows matching the position of the primary tumour during treatment delivery with the treatment-planning CT scan, including adaptation to changes including movements and tumour growth or shrinkage.

The next level of IGRT followed the introduction of the MRI-LINACs, facilitating anatomical/geographic position verification and functional imaging, allowing for adaptation of the dose distribution within the target volume [226, 227]. Similarly, recently proposed as a new concept, the combination of a PET with a linear accelerator offers advantages by generating biological/functional information, ambitiously called biology-guided RT (https://reflexion.com/). The PET/LINAC combination can visualize and subsequently deliver treatment to multiple cancer sites during one single session.

Currently, the physical integration of a linear accelerator with either MRI or PET into one single unit is challenged by an optimized sequential workflow of functional and anatomical imaging on separate machines [228, 229]. The scientific question to be resolved became thereby whether simultaneous or sequential functional imaging will be superior. Likely, this may depend on the indication, depending on factors such as tumour biology and mobility.

### Positron emission mammography (PEM) and dedicated breast PET (dbPET)

There are several dedicated breast PET systems that make use of breast compression, also known as positron emission mammography (PEM), and ring-shaped scanners for imaging uncompressed hanging breasts in the prone position, known as dedicated breast PET systems (dbPET). These systems visualize 2-[^18^F]FDG uptake or other tracer uptake in a small field of view. The advantages of such systems over whole-body PET/CT are their higher spatial resolution (1.6 mm), shorter imaging time, reduced attenuation, and higher count sensitivity, allowing PET-guided biopsy and resulting in a higher sensitivity to detect primary breast malignancies than whole-body 2-[^18^F]FDG PET/CT (identification rate of 95% vs. 87%). The diagnostic performance of PEM/dbPET is higher than that of X-ray mammography or US, and comparable to that of MRI in the identification of invasive BC. Furthermore, its diagnostic performance is not affected by dense breast tissue. Due to the limited field of view, PEM/dbPET can miss small deeply located lesions closer than 2 cm to the chest wall, next to the pectoral muscle, or in the axillary region [230, 231].

### Metastasis-directed treatment in oligometastatic breast cancer

2-[^18^F]FDG PET/CT is useful for RT planning in patients with oligometastatic disease, particularly to exclude other metastatic sites prior to curative intent radioablation [232–235].

Currently, there exist no validated biomarkers for response evaluation after ablative RT in patients with oligometastatic disease, particularly in the context of BC. Nevertheless, a cohort study published in 2012 concluded that 2-[^18^F]FDG PET/CT performed 1.2 months post-therapy enabled response monitoring to SBRT in non-measurable metastases by CT and it could be used as a potential imaging biomarker [235]. 2-[^18^F]FDG PET/CT has the advantage of identifying oligometastatic disease resistant to treatment at a very early phase, allowing for locoregional ablative treatment which often offers a favourable clinical impact [232].

With the goal of optimizing surveillance imaging protocols, a joint initiative between the ESTRO and the EORTC has launched a prospective, large-scale observational study for oligometastatic patients entitled Oligocare (NCT03818503). The preliminary results suggest that 2-[^18^F]FDG PET/CT is favoured in BC (with wbMRI or PET/MR as alternatives) but needs to be supplemented by liver-specific MRI [232, 236]. The final conclusions of these clinical trials will improve the understanding of this topic.

### Image analysis and quantification

Textural features that measure tumour heterogeneity and changes in the surrounding stroma have also emerged as potential prognostic imaging biomarkers in BC studies [237, 238]. Nevertheless, despite encouraging results, studies are far from providing definitive conclusions. This is mainly due to variations in acquisition, reconstruction, segmentation, and radiomic processing between studies, which makes its clinical application challenging [239]. This underlines the importance of using harmonization programs (e.g. EARL certification). Furthermore, radiomics analysis should also follow the definitions of the Image Biomarker Standardization Initiative (IBSI) [240].

Currently, there is no consensus regarding the optimal metabolic parameter and time to perform 2-[^18^F]FDG PET/CT for response monitoring [58]. The optimal timing for PET/CT imaging depends on tumour phenotype and therapeutic schemes as well as on local protocols and reimbursement considerations [58, 137, 141].

A systematic review from 2019 evaluated response to first- or second-line systemic therapy in patients with metastatic BC [158]. The authors highlighted one study that compared the use of RECIST (with ceCT) and PERCIST (with 2-[^18^F]FDG PET/CT) and demonstrated that 40% of non-responding tumours based on RECIST were responders on PERCIST criteria, demonstrating that metabolic assessment with 2-[^18^F]FDG PET/CT may be a better predictor of both progression-free and disease-specific survival than CT in patients with metastatic BC [157, 158]. Despite the extensive literature on the use of (semi)quantitative PET parameters in oncology, specific studies about BC are lacking.

### Supplementary information

Below is the link to the electronic supplementary material.

Supplementary file1 (DOCX 51.1 KB)

## Data Availability

The datasets generated during and/or analyzed during the current document are available from the corresponding author on reasonable request.

## Declarations

### Endorsements

This guideline has been reviewed and endorsed by the following societies: American College of Radiology (ACR), European Society of Surgical Oncology (ESSO), European Society for Radiotherapy and Oncology (ESTRO), European Society of Breast Imaging/ European Society of Radiology (EUSOBI/ESR), and European Society of Breast Cancer Specialists (EUSOMA).

### Liability statement

This guideline summarizes the views of the EANM Oncology and Theranostics Committee and SNMMI. It reflects recommendations for which the EANM and SNMMI cannot be held responsible. The recommendations should be taken into the context of good practice of the different disciplines (mainly Nuclear Medicine, Breast Radiology, Radiation Oncology, Medical Oncology, and Breast Surgery) and do not substitute for national and international legal or regulatory provisions.

## Abbreviations

2-[^18^F]FDG: 2-[^18^F]Fluoro-2-deoxy-D-glucose
BC: Breast cancer
CA15.3: Cancer antigen 15.3
CA125: Cancer antigen 125
CEA: Carcinoembryonic antigen
ceCT: Contrast-enhanced CT
CT: Computed tomography
EANM: European Association of Nuclear Medicine
ER: Oestrogen receptor
ESMO: European Society of Medical Oncology
ESTRO: European Society for Radiotherapy and Oncology
HIF-1a: Hypoxia-inducible factor 1-alpha
HR + or HR: Hormone (oestrogen or progesterone) receptors present or absent
IDC: Invasive ductal carcinoma
IGRT: Image-guided radiation therapy
ILC: Invasive lobular carcinoma
IMRT: Intensity-modulated radiation therapy
MTV: Metabolic tumour volume
MRI: Magnetic resonance imaging
NCCN: National Comprehensive Cancer Network
OS: Overall survival
PEM: Positron emission mammography
PET: Positron emission tomography
PPV: Positive predictive value
PR: Progesterone receptor
RCT: Randomized controlled trials
ROC: Receiver operating characteristic
RT: Radiation therapy
SBRT: Stereotactic body radiation therapy
SEER: Surveillance, Epidemiology, and End Results Program
SNMMI: Society of Nuclear Medicine and Molecular Imaging
SULmax: SUVmax corrected for lean body mass
SUVmax: Maximum standardized uptake value
TLG: Total Lesion glycolysis
TNBC: Triple-negative breast cancer
tSRE: Time to skeletal-related events
TTP: Time to progression
US: Ultrasonography
wbMRI: Whole-body MRI
