# Supplementary material for: Joint EANM-SNMMI guideline on the role of 2-[18F]FDG PET/CT in no special type breast cancer: (endorsed by the ACR, ESSO, ESTRO, EUSOBI/ESR, and EUSOMA)
Source: Eur J Nucl Med Mol Imaging. 2024 May 14;51(9):2706–32. doi: 10.1007/s00259-024-06696-9 (PMC11224102; doi:10.1007/s00259-024-06696-9)
Supplement: Supplementary file 1 — Supplementary file1 (DOCX 51.1 KB) [file 259_2024_6696_MOESM1_ESM.docx]

**SUPPLEMENTARY INFORMATION**

The European Association of Nuclear Medicine (EANM) is a professional non-profit medical association that facilitates communication worldwide between individuals pursuing clinical and research excellence in nuclear medicine. The EANM was founded in 1985. The Society of Nuclear Medicine and Molecular Imaging (SNMMI) is an international scientific and professional organization founded in 1954 to promote the science, technology, and practical application of nuclear medicine. The EANM and SNMMI members include physicians, radiologists, technologists, and scientists specializing in the research and practice of nuclear medicine.

The EANM and SNMMI periodically define new guidelines for nuclear medicine practice to help advance the science of nuclear medicine and improve the quality of service to patients throughout the world. Existing practice guidelines are reviewed for revision or renewal, as appropriate, on their fifth anniversary or sooner, if indicated. Each practice guideline, representing a joint policy statement by the EANM/SNMMI, has undergone a thorough consensus process in which existing evidence has been subjected to extensive review. The EANM and SNMMI recognize that the safe and effective use of diagnostic nuclear medicine imaging requires specific training, skills, and techniques, as described in each document. Reproduction or modification of the published practice guideline by those entities not providing these services is not authorized.

These guidelines represent an educational tool designed to assist practitioners in providing appropriate care for patients. They are not inflexible rules or requirements of practice and are not intended, nor should they be used, to establish a legal standard of care. For these reasons, and those set forth below, both the EANM and the SNMMI caution against the use of these guidelines in litigation in which the clinical decisions of a practitioner may be called into question.

The ultimate judgment regarding the propriety of any specific procedure or course of action must be made by the physician or medical physicist in light of all the circumstances presented. Thus, there is no implication that an approach differing from the guidelines, standing alone, is below the standard of care. To the contrary, a conscientious practitioner may responsibly adopt a course of action different from that set forth in the guidelines when, in the reasonable judgment of the practitioner, such course of action is indicated by the condition of the patient, limitations of available resources, advances in knowledge or technology subsequent to publication of the guidelines, local regulatory requirement, or reimbursement frameworks. The practice of medicine includes both the art and the science of the prevention, diagnosis, alleviation, and treatment of disease. The variety and complexity of human conditions make it impossible to always reach the most appropriate diagnosis or to predict with certainty a particular response to treatment.

Therefore, it should be recognized that adherence to these guidelines will not ensure an accurate diagnosis or a successful outcome. All that should be expected is that the practitioner will follow a reasonable course of action based on current knowledge, available resources, and the needs of the patient to deliver effective and safe medical care. The sole purpose of these guidelines is to assist practitioners in achieving this objective.

Members (and ex-members) of the EANM Oncology Committee (Sofia C. Vaz, Patrick Pilkington and Lioe-Fee de Geus-Oei) and the SNMMI representatives (Heather Jacene and Elizabeth Dibble), invited a multidisciplinary panel of European and American experts from the disciplines of Nuclear Medicine (David Groheux, Gary Cook and Gary Ulaner), Breast Radiology (Ritse Mann), Radiation Oncology (Philip Poortmans), Medical Oncology (Fátima Cardoso and Stephanie Graff), and Breast Surgery (Isabel-Teresa Rubio and Marie-Jeanne Vrancken Peeters) to take part in developing this guideline.
